# Major concerns about late hypothermia study

**DOI:** 10.1111/apa.14640

**Published:** 2018-11-28

**Authors:** Lars Walløe, Nils Lid Hjort, Marianne Thoresen

**Affiliations:** ^1^ Division of Physiology Institute of Basic Medical Sciences University of Oslo Oslo Norway; ^2^ Division of Statistics and Biostatistics Department of Mathematics University of Oslo Oslo Norway; ^3^ Neonatal Neuroscience Translational Health Sciences University of Bristol Bristol United Kingdom

## Abstract

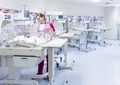

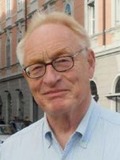

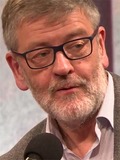

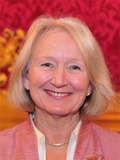

An important neonatal hypothermia (HT) trial on hypoxic–ischaemic encephalopathy was published in November 2017 by Laptook and the NICHD group 1. One hundred and sixty‐eight newborn term infants who failed the six‐hour time‐window for HT were recruited by 21 centres over an eight‐year period into a trial of HT versus normothermia (NT) if they were more than six hours and less than 24 hours old. The median postnatal age at start of HT was 16 hours. The infants were cooled for 96 hours, 24 hours longer than the standard 72 hour duration. Survivors were examined at 18–22 months using the Bayley Scales of Infant Development III and a neurological examination. Outcome data were available for 157 infants including 18 who died (78 HT–19 with poor outcome, 79 NT–22 with poor outcome). Clearly, any outcome difference between the HT and NT groups must be small, and not significant (p ≈ 0.75). However, the authors conclude, based on Bayesian analyses, that ‘among term infants with hypoxic‐ischemic encephalopathy hypothermia initiated at 6–24 hours after birth compared with noncooling resulted in a 76% probability of any reduction in death or disability’.

In an EBNEO commentary in Acta Paediatrica 2, Bourque and Dietz argue that the ‘potential benefit purported by this trial should prompt NICUs to consider initiation of TH beyond six hours of age’. However, our judgement is that the results from the JAMA paper do not support the strong conclusions presented by Bourque and Dietz. Recently, the suggestion to allow late cooling (>6 and <24 hours) was implemented in an ongoing randomised trial of treating mild HIE with cooling at 33.5°C 3.

In Bayesian analyses, the probability of a treatment effect (the posterior probability distribution) is estimated after the trial has been carried out and incorporates the prior probability distribution estimated from data from previous studies if such data are available. In the present case, there is no available data from trials or pilot studies in human infants with time of recruitment less than six hours postpartum. Most of the results in the paper are presented from an analysis using a ‘neutral’ prior (assuming no treatment effect, relative risk (RR) = 1.0). However, even with an RR = 1.0, the authors have the possibility to choose freely both the shape and the width of the prior distribution. They have chosen a normal distribution on a log scale with SD = 0.35. This choice of distribution is not ‘non‐informative’ in the Bayesian sense.

However in the present case, there is additional relevant prior information from experimental studies in animals (foetal sheep 4 and seven day neonatal rats 5). The information obtained from these studies is very clear and similar in the two very different species. The therapeutic effect of cooling diminishes linearly with the time of start of cooling and is zero after nine hours postinsult. The results presented by Laptook et al. 1 support that the findings from the two animal species of no effect of late cooling are also valid for newborn humans. In addition, an observational human cooling study 6 showed that starting cooling between zero and three hours resulted in better motor outcome at 18 months than those who started cooling between three and six hours. There is also a concern that both human and experimental data suggest that longer cooling may be harmful 7, 8. In the foetal sheep experiments, cooling for 120 hours was less protective than cooling for 72 hours 8.

When Laptook et al. provide probabilities for the relative risk parameter RR = p_1_/p_0_ being less than 1, or less than 0.98, etc., these are computed inside a Bayesian framework, and influenced by the prior. When using a standard non‐informative Jeffreys prior, the 95% credibility interval for RR becomes [0.53, 1.53], of course containing the null value RR = 1. With a non‐Bayesian analysis, RR is an unknown parameter, and one cannot assign any clear probability to statements like RR ≤ 0.98. We may, however, construct a full confidence curve, as in Schweder and Hjort 9. Figure 1 shows this curve, effectively providing confidence intervals at all RR (p_1_/p_0_) levels. The 95% interval is [0.51, 1.48], agreeing well with the default Bayes. All intervals of level 38% or more contain the null hypothesis value RR = 1.00; there is hence no reason to reject that value. The main conclusion from the Laptook et al. study 1 ought to be that there is simply no real difference between the therapeutic probabilities for the two groups. In addition, with a normal prior as in Laptook et al. 1, the posterior distribution should be skewed with a tail to high RR values, not normal as in their figure 2.

**Figure 1:**
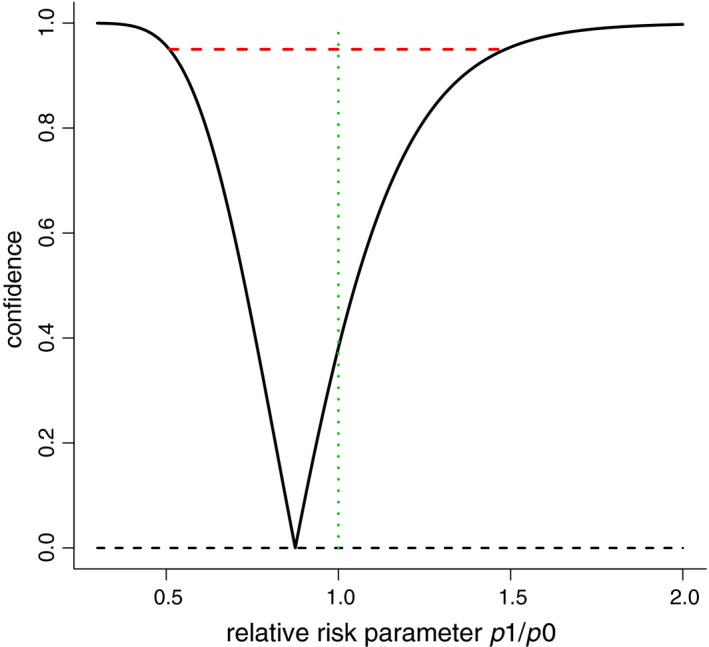
Confidence probability (*y*‐axis) for the relative risk parameter RR = (p_1_/p_0_) (*x*‐axis) points to the estimate 0.875 and provides confidence intervals at all levels of confidence probability. The 95% confidence interval is [0.51, 1.48] (broken horizontal line).

It is our view that one of the main conclusions of the JAMA paper 1, that ‘The probability that death or disability in cooled infants was at least 1%, 2%, or 3% less than noncooled infants was 71%, 64% and 56%, respectively’ when HT was initiated 6–24 hours after birth, is probably wrong, at least highly speculative and should not be used as an argument for change to current cooling regimens.

A potential danger connected to this paper is that the current strong requirement that babies should be cooled as soon as possible after birth will be relaxed.

## Conflict of interests

The authors have no conflict of interests.

## Funding

Funding supporting work on therapeutic hypothermia was received by the Norwegian Research Council and the Medical Research Council, UK.
